# A Doppler Radar System for Sensing Physiological Parameters in Walking and Standing Positions

**DOI:** 10.3390/s17030485

**Published:** 2017-03-01

**Authors:** Malikeh Pour Ebrahim, Majid Sarvi, Mehmet Rasit Yuce

**Affiliations:** 1 Department of Electrical and Computer Systems Engineering, Monash University, Clayton, Melbourne 3168, Australia; melika.pour.ebrahim@monash.edu; 2 Department of Infrastructure Engineering, Melbourne School of Engineering, The University of Melbourne, Melbourne 3010, Australia; msarvi@unimelb.edu.au

**Keywords:** Doppler radar, physiological parameters monitoring, arctangent demodulation, respiration rate, remote sensing

## Abstract

Doppler radar can be implemented for sensing physiological parameters wirelessly at a distance. Detecting respiration rate, an important human body parameter, is essential in a range of applications like emergency and military healthcare environments, and Doppler radar records actual chest motion. One challenge in using Doppler radar is being able to monitor several patients simultaneously and in different situations like standing, walking, or lying. This paper presents a complete transmitter-receiver Doppler radar system, which uses a 4 GHz continuous wave radar signal transmission and receiving system, to extract base-band data from a phase-shifted signal. This work reports experimental evaluations of the system for one and two subjects in various standing and walking positions. It provides a detailed signal analysis of various breathing rates of these two subjects simultaneously. These results will be useful in future medical monitoring applications.

## 1. Introduction

Noncontact detection and monitoring of human respiration as a non-invasive procedure has been explored for many years [1]. Such a method, if it is reliable and stable, can be used in a range of applications such as emergency and military healthcare settings [2,3]. It can be part of a low-power medical sensor network system in medical centres for collection and monitoring of certain physiological parameters. In most cases, breathing rate-monitoring techniques require contact between the person and the equipment (like electrocardiogram, stethoscope, oximeter, or phono-cardiogram), which may cause discomfort for the person, while wireless recordings can be made without any physical contact [1]. A radar-based sensing method does not require physical contact with patients and also presents an opportunity to monitor several patients simultaneously [1]. The performance of a Doppler radar system depends on various factors, such as body posture, body movement, absorption characteristics of the human body at radar frequency, and antennas’ characteristics [4]. A transceiver radar system should be designed to operate reliably under such conditions.

Recently, there has been increasing interest from researchers, system designers, and application developers on medical network architectures generally known as body sensor networks (BSNs) or body area networks (BANs), using lightweight, small-size, ultra-low-power, and intelligent wearable sensors for monitoring of important physiological signals [5]. A wireless body sensor network is a collection of wearable (programmable) sensor nodes communicating with a local personal device (also known as body control device) [6]. The proposed noncontact sensing technology can be implemented within the body control device of a BAN system or attached to the body in the form of a wearable sensor among other wireless wearable sensors to form an intelligent BSN and BAN for medical monitoring applications.

Noncontact physiological radar monitoring systems have been used efficiently to perform sleep monitoring. For example, they were used in [7] to categorise normal breathing, apnoea, and hypopnea in sleeping subjects with obstructive sleep apnoea. The designed system was mounted on a vertical stand and was directed to face the chest of supine subjects, but was not optimised for other directions.

Typically, a Doppler radar motion-sensing transceiver transmits a continuous wave signal, which is reflected off a target and demodulated in the receiver [8]. A human body has two independent sources of motion caused by respiration and heart activities. The frequency of human breathing can range anywhere from 0.1 to 0.5 Hz [9]. Data in the receiver will be a phase-shifted signal, which will include respiration information [10]. A field-programmable gate array (FPGA)-based system was designed and implemented to detect human heartbeat and respiration using Doppler processing. The system generated an ultra-wideband (UWB) stepped frequency continuous wave of bandwidth of 3 GHz. For the experimental setup in this research, a human at a position 0.55 m behind a barrier was considered as the target [9].

One challenge in providing robust sensing is the detection sensitivity due to the periodic phase relationship between the received signal and local oscillator (LO), resulting in “optimum” and “null” extreme target positions [2]. A combination of quadrature outputs using arctangent demodulation with DC offset compensation was proposed to solve this problem [2,11]. A Doppler radar system based on phase diversity was used to overcome null detection. It utilises a two-channel receiver with a convenient phase-shift between channels that ensures proper detection [8,12]. Also, a quadrature Doppler radar receiver with channel selection was applied, which selects the better quadrature (I and Q) channel outputs [13].

In [2], the problem of extracting the delicate heartbeat and breathing rate modulation from the stationary part of the signal is addressed. However, such a DC information component, associated with target position in Doppler radar, is typically several orders of magnitude greater than the amplitude of the periodic baseband signal related to heart and breathing activity [2]. Therefore, the simplest solution to remove DC offsets is to use a high-pass filter.

Table 1 compares some recent research that used different systems and methods to measure respiration rate and heartrate. 

Studies in [3,18] did not use real subjects to evaluate their systems, but just simulated biomedical signals. The systems developed by [2,10,11,15,16,17] were used to measure vital signs, but only in lying or seated positions. The experiments in [4,19,20,21] were done in standing and walking positions; however, only one subject took part in each test.

While most research used continuous-wave (CW) Doppler radar for monitoring heartbeat and breathing rates, lots of them chose standard 2.4 GHz for carrier frequency, as in [2,3,7,8,15,19,20,21], and some used standard 5.8 GHz, as in [16,17].

A further characteristic which is compared in Table 1 is the potential of the systems to detect vital signs at different distances. While most research using CW radar works on just a single distance, this work was set up and used successfully for three different distances.

While many publications present the implementation of a radar-based system for just one person, very few people investigate whether such a system has the ability to detect the breathing rate of more than one person. This work presents a single, less-complex setup with beamforming or antenna arrays, to compare respiration signals of one or two people at the same time at distances from 30 cm to 150 cm, and at different angles in front of antennas, to determine if the system can recognise two different breathing rates. On the other hand, most previous designs have not considered the complications created by subjects while walking. One important issue in recording breathing data is getting signals from people in typical life conditions such as walking. For people who are just sitting or standing in traditional testing situations, the signal being recorded will not be distorted. In this research, we try to show how the setup can get proper data for a person who is walking for a distance between 30 cm and 150 cm in front of an antenna and from different angles.

This work reports a complete transmitter-receiver radar system, which uses 4 GHz CW for data transmission and a mixer to extract base-band signal. A complete platform including, a signal transmitter and a receiver, antennas, a mixer, and data-processing software, has been implemented and evaluated for respiration signals. This paper presents experimental evaluation of the system for one and two subjects in various standing and walking positions.

The rest of the paper is organised as follows: Section 2 presents the method including hardware and software experiment setup and human testing for different protocols. Section 3 describes experimental results of the system, and Section 4 concludes the paper.

## 2. Method

### 2.1. Experiment Setup

**Figure 1:**
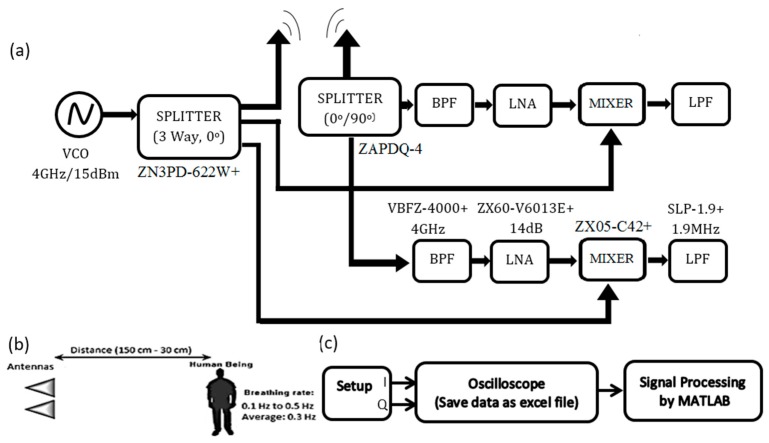
Radar system for remote sensing: (**a**) block diagram of the setup; (**b**) antennas and subject position for tests; (**c**) block diagram of the whole process.

The experiment’s setup, is shown in Figure 1. Radar system for remote sensing: (a) The block diagram of the setup; (b) antennas and subject position for tests; (c) a block diagram of the whole process. The setup includes three main components: (1) two transmitter and receiver radar antennas; (2) the amplifiers, band-pass and low-pass filters and splitters; and (3) the data acquisition and signal-processing system (using an oscilloscope and MATLAB). The setup was configured with two In-phase and quadrature channels. Then, the arctangent demodulation was performed on these I and Q signals using the MATLAB protocols.

Radar recording heartbeats and respiration from a distance does not only record the chest moving, but also all other objects within the antenna’s beam as DC offset [11]. This DC offset results from the receiver’s imperfections and clutter reflections, in addition to DC information related to the target’s position and its associated phase, and can be significant compared to the AC motion-related signal [15]. If *T*(*t*) is the transmitted signal and *R*(*t*) is the received signal after antennas, the following formulae result:

(1) $$T\left(t\right)=E_{T}\;cos\left(\omega t+\varphi \right),$$

(2) $$R\left(t\right)=E_{R}\cos\left(\omega \left(t-t_{d}\right)+\theta \right),$$

(3) $$t_{d}=(d_{0}+m\left(t\right))/c=2\left(d_{0}+m_{r}\left(t\right)\right)/\lambda f,$$

(4) $$R\left(t\right)=E_{R}\cos\left(\omega t-4\pi d_{0}/\lambda -4\pi m_{r}\left(t\right)/\lambda +\theta \right),$$

where:

(5) $$m_{r}\left(t\right)=\cos\left(\omega_{r}t\right).$$

*E_T_* is the amplitude of transmitted signal, *E_R_* is the amplitude of received signal, *t_d_* is the delay time, θ is the phase shift related to DC offset, d_0_ is the distance between target and antenna, *m_r_*(*t*) is chest movement caused by respiration, and λ is wavelength of the carrier signal.

The quadrature components are as follows:

(6) $$I\left(t\right)\approx \cos\left(4\pi d_{0}/\lambda +4\pi m_{r}\left(t\right)/\lambda +\theta \right).$$

(7) $$Q\left(t\right)\approx sin\left(4\pi d_{0}/\lambda +4\pi m_{r}\left(t\right)/\lambda +\theta \right).$$

(8) $$\varphi \left(t\right)=\tan^{-1}Q\left(t\right)/I\left(t\right)=4\pi d_{0}/\lambda +\left(4\pi m_{r}\left(t\right)/\lambda +\theta \right).$$

where φ(t) is the phase change caused by chest movement. φ(t) contains DC information related to target position, which can be removed, therefore, accurate phase demodulation will be obtained regardless of the target position [2]. The tests were performed in five categories on each subject. Two measurements were taken while the subject was standing. Two other tests were performed while subject was walking, and the last measurement was for the two subjects standing while facing an antenna at the same distance. Figure 1b shows the position of the antennas and the subject in different situations.

### 2.2. Hardware

**Figure 2:**
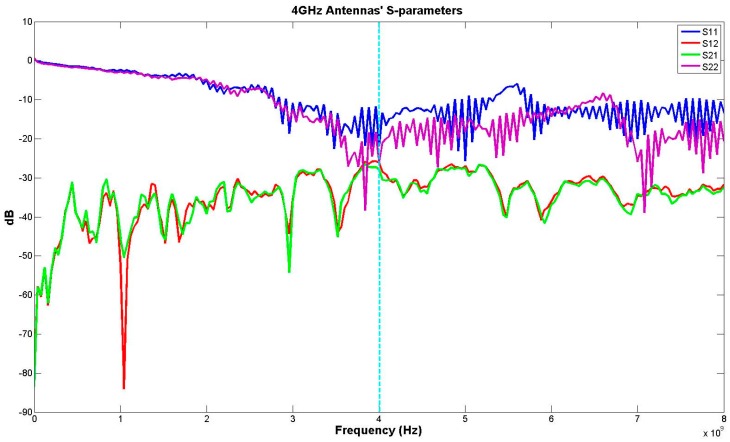
Antennas’ S-parameters.

Figure 1 shows the block diagram of the radar system, which includes two main transmitter and receiver parts. A generated CW signal is transmitted by an antenna. Antennas used are designed for wideband communications and operate within 3–10 GHz range and are non-directional. Figure 2 shows antennas’ S-parameters in their frequency range. Directional antennas would enable signal detection for a longer distance. For a non-directional antenna, transmitter power or the gain at the receive chain can be increased to obtain a similar distance. In our experiment, as we were interested in positions in a wider space (e.g., different positions/angles to the antennas) to be detectable, an antenna with wider beam width is preferable. In case of directional antenna, in order to detect multiple people, multiple antenna arrays will be a better option. 

On one hand, a voltage control oscillator (VCO-Minicircuit/ZX95-4040+) can be used to generate the transmitted signal, which is delivered at 4 GHz. On the other hand, a signal generator (ROHDE & SCHWARZ—SMA 100 A) can be used instead of a VCO to generate the signal with the ability of controlling the signal amplitude. A single signal source was used to provide both the radiofrequency (RF) output and LO signals. The generated signal was transmitted through the setup with a three-way splitter (Minicircuit/ZN3PD-622W-S+). One output of the splitter connects to a transmitter antenna and two other outputs connect to two mixers in the receiver part of the setup.

**Figure 3:**
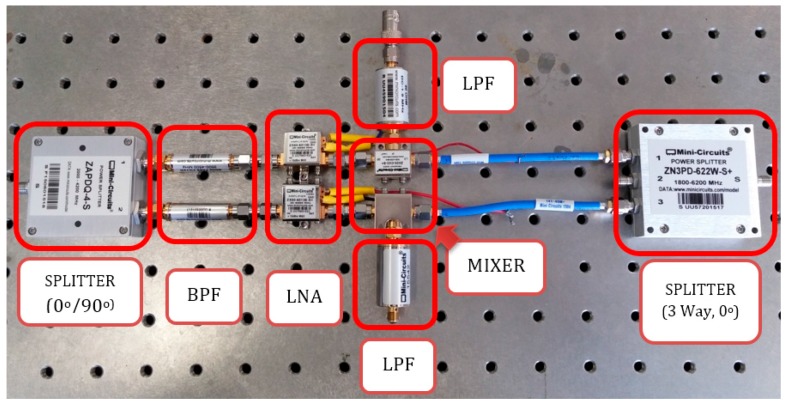
Setup components and connections.

The received signal from the receiver antenna is converted to two I and Q components (In phase and quadrature) by a two-way 0o/90o splitter (Minicircuit/ZAPDQ-4–S). These signals, after being passed from two band-pass filters (Minicircuit/VBFZ-4000+/4 GHz) and amplified by two low-noise amplifiers, (LNA-Minicircuit/ZX60-V6013E+/14 dB) connect to the mixers. The output signals of the mixers will be two I and Q components of a base-band signal and include respiration information. To remove the high-frequency noises from the signals, two low-pass filters (LPF-Minicircuit/SLP-1.9+/1.9 MHz) are placed after the mixers. Figure 3 shows details of the different components and connections of the setup.

### 2.3. Software

#### 2.3.1. Data Acquisition

**Figure 4:**
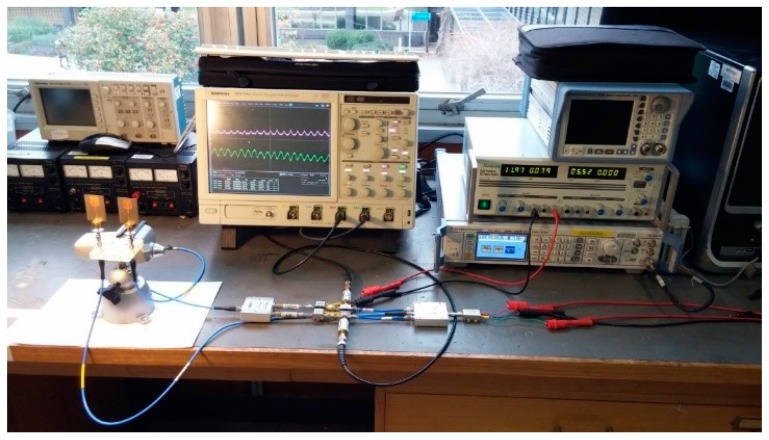
Experimental setup.

For data acquisition from the radar system, a MATLAB program was developed. To record and analyse the signals, the outputs of the LPFs were connected to two channels of an oscilloscope (Tekronix) as shown in Figure 4. Two I and Q channels of data were recorded simultaneously. The data were sampled at 500 S/s and stored on a memory card as an Excel file. Then, the recorded data were loaded to a MATLAB workspace by an M.file to analyse and perform feature extraction.

First the DC level of I and Q signals was eliminated by removing their mean value. Then, they were passed through different filtering levels to gain each subjects’ data. The first filtering level was a 40th-order finite impulse response (FIR) low-pass filter with a cutoff frequency of 5 Hz and gain settings to obtain a sharp cutoff. To have a maximum ratio of main lobe energy to side lobe energy, a Kaiser window was used to design the LPF. Then a 40th-order FIR high-pass filter (HPF) with a cutoff frequency of 0.05 Hz was applied to remove DC noise and very-low-frequency noise components. Again, a Kaiser window was used for designing HPF. Finally, a 2500th-order FIR-band-pass filter (BPF) with pass band of 0.1–0.5 Hz, based on the frequency of human breathing [9], was used. The order of BPF was selected as 2500 to have a high resolution (in desired) for the low-frequency band. Moreover, two digital IIR notch filters with frequencies of 0.01 Hz and 0.05 Hz were considered to eliminate two noisy high amplitude frequency components. The reason an LPF was used for the first stage of filtering was to avoid unwanted high-frequency components and, specifically, a 50 Hz power line. On the other hand, HPF and two notch filters were used to eliminate unwanted very-low-frequency components. Since these components have high amplitude in comparison with breathing rate amplitude, and this affects detection of respiration rate, one level BPF with a pass band of 0.1–0.5 Hz was not enough, and different LPF, HPF, and notch filters were used to weaken unwanted frequency components as much as possible.

After filtering levels, using fast Fourier transforming (FFT), the frequency domain of each I and Q component was calculated to find the frequency of respiration based on time domain signals. For this purpose, the discrete Fourier transformation (DFT) of signal using an FFT algorithm was computed. In order to improve the performance of FFT, a new length of data, as n, that was the next power of two from the original signal length, was used to have an n-point DFT.

For spectral peak detection, the maximum peak observed in the frequency domain of the signal in an acceptable frequency range related to respiration was selected as the calculated respiration or heartrate.

#### 2.3.2. Arctangent Demodulation

The setup provides two orthonormal outputs, which means that when one channel is in a “null” position the other will be in an “optimum” position [2]. On one hand, it is possible to select the better of the quadrature (I and Q) channel outputs, so the accuracy of results will be limited to a single channel [2]. On the other hand, by applying arctangent demodulation to the I and Q channels’ outputs, an accurate phase demodulation can be achieved regardless of the target position or displacement amplitude [2]. Based on Equations (1) and (2), the I and Q outputs are the cosine and sine of a constant phase delay caused by the nominal distance to a target with a time varying phase shift that is linearly proportional to the chest displacement [2]. By applying the arctangent operation to the I and Q ratio, phase demodulation will be as Equation (8).

**Figure 5:**
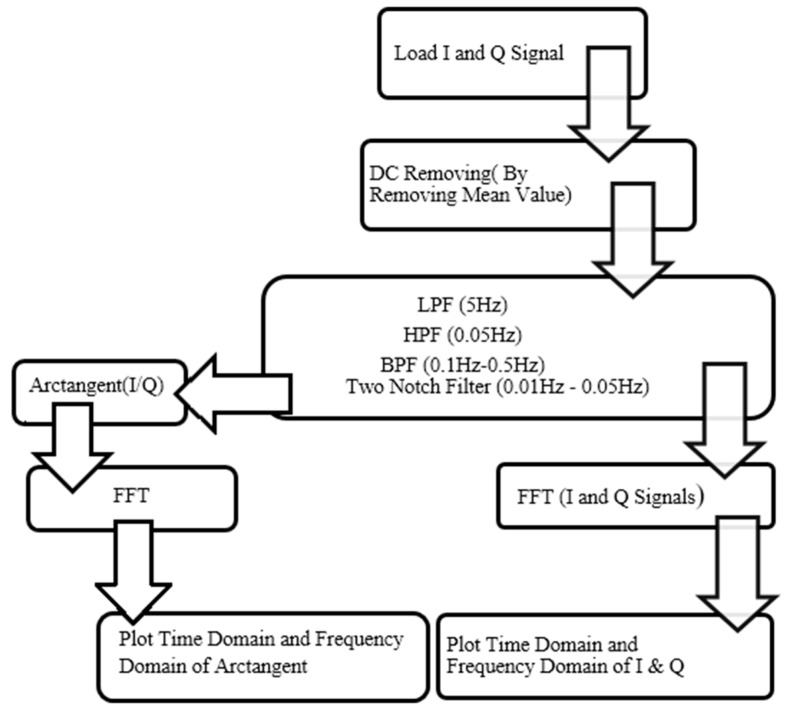
The block diagram of different levels of signal processing using MATLAB.

Arctangent demodulation was calculated using MATLAB functions, then the frequency domain was extracted by performing FFT. An FFT algorithm, the same as the one used for I and Q channels, was applied to the time domain arctangent result. Figure 5 shows the block diagram of different levels of signal processing used.

### 2.4. Experimental Protocol and Human Testing

Eight healthy subjects without any reported breathing problems volunteered to participate in the tests. The data were taken from volunteers in different positions during walking and standing. The subjects wore normal clothing and were asked to breath normally during the measurements.

**Figure 6:**
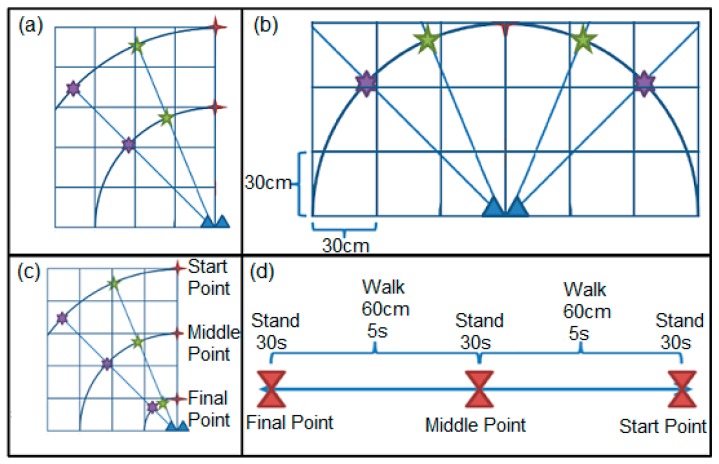
The positions of tests protocols: (**a**) the places of standing for tests 1 and 2; (**b**) the positions of standing for two people in test 3; (**c**) the directions of walking for tests 4 and 5; (**d**) the directions of walking and the places of standing during walking for test 5.

As shown in Figure 6, different situations for recording respiration signals were as follows:

1. Each target person stood at two different distances (90 cm and 150 cm), and three different angles (0°, 22.5°, and 45°), while facing the antennas and breathed normally for 50 s.
2. Each target person stood at two different distances (90 cm and 150 cm) while not facing the antennas and breathed normally for 50 s (0° direction).
3. Two subjects stood at the same distances (90 cm and 150 cm) and the same angles (22.5° and 45°) and breathed normally and simultaneously for 50 s to understand if the setup could realise different respiration rates.
4. Each subject walked a distance of between 30 cm and 150 cm and in three different directions (0°, 22.5°, and 45°) for 50 s at a constant speed, without standing still, to find out whether or not the setup could measure the respiration rate during walking.
5. The position of test protocols:
  - At first, each subject stood at a distance of 150 cm from the antennas for 30 s, and then walked at a distance of between 150 cm and 90 cm from the antennas for 10 s.
  - Afterward, the subject stood at a distance of 90 cm from the antennae for 30 s, and then walked at a distance of between 90 cm and 30 cm from the antennas in 10 s.
  - Finally, the subject stood at a distance of 30 cm from the antennas for 30 s.

The field of view in Figure 6 is basically due to the fact that an antenna with a wider beam width is used. To obtain the ground-truth for subjects’ breathing rates, it was requested that each subject count his/her breathing inhales and exhales during tests, and these data were used as the reference for respiration rate value. In future, peak detection algorithms can be implemented in MATLAB with a threshold providing the best signal-to-noise ratio for autonomous detection of breathing rate.

## 3. Experimental Results and Discussion

**Figure 7:**
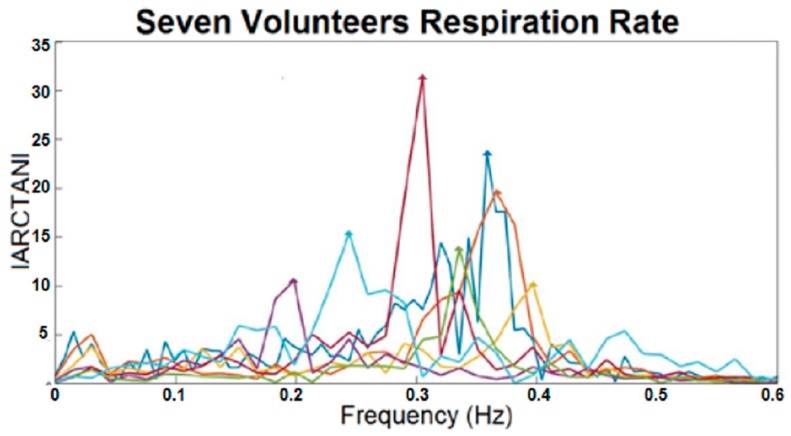
Frequency domain of I, Q, and arctangent demodulated signals for seven subjects at the same distance from the antennas and facing the same direction.

For seven volunteers, the results of standing at the same distance from the antennas and facing the same direction are shown in Figure 7. The respiration frequency of all subjects is in Table 2. It should be noted that the tests were done for each person separately. The arctangents of the Q/I ratio in the frequency domain express that, besides the difference between frequencies of breathing for subjects, the amplitude of chest movements for them also vary. As a result, it can be said that respiration rate can be used as a factor of identifying people.

**Figure 8:**
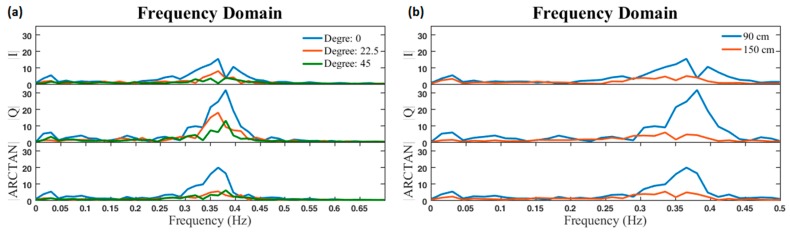
Frequency domain of I, Q, and arctangent demodulated signals. (**a**) Signals of a standing subject at the same 90 cm distance from the antennas and facing in different directions; (**b**) signals of a standing subject at two different distances from the antennas and facing the same direction (0°).

For one subject, the results of test 1 are shown in Figure 8. The I and Q outputs and the arctangent of the Q/I ratio are shown in time and frequency domains. Based on Figure 8, the breathing rate of the subject is clearly visible. Figure 8a illustrates the effect of various directions proportional to the position of antennas for the same 90 cm distance. The graphs express that, if a person stands in a direction perpendicular to the antennas’ plane, in comparison with other directions, the amplitude of signals will be maximised. This is because of the antennas’ characteristics. Figure 8b indicates how different distances affect the amplitude of signals.

**Figure 9:**
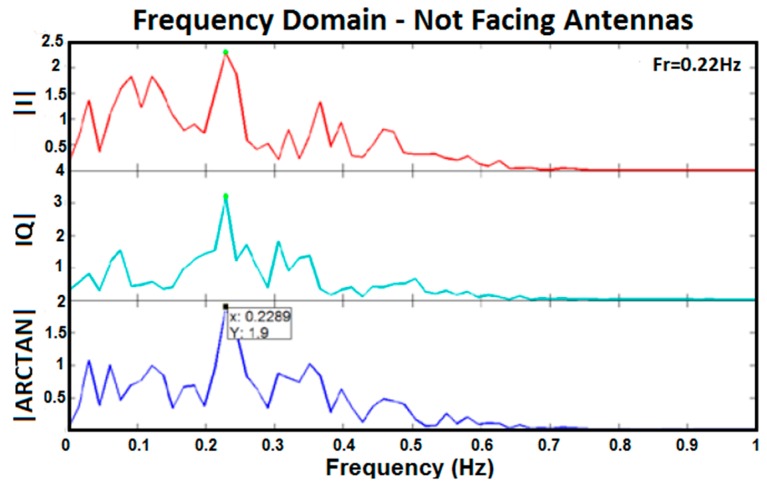
Frequency domain of I, Q, and arctangent demodulated signals for a subject not facing the antennas.

The results of test 2 are shown in Figure 9. The figure shows the I, Q, and arctangent of the Q/I for a subject who stood in front of antennas while not facing them. Compared with result of facing the antennas, the figure indicates the signals have more noise. This means that it is almost impossible to find the frequency of respiration based on a time domain diagram. However, when different levels of signal processing based on the block diagram in Figure 5 were applied to the signal, it was still noisy, and finding the accurate and reliable frequency of respiration was more challenging. One reason was that regular movement of the chest related to breathing was more obvious while facing antennas in comparison with not facing them.

**Figure 10:**
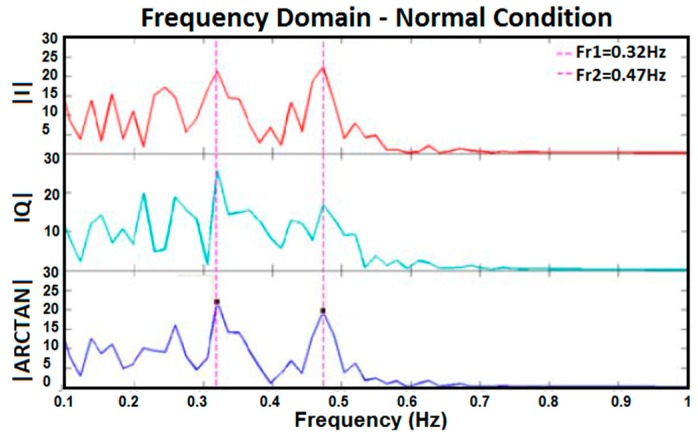
Frequency domain of I, Q, and arctangent demodulated signals for two subjects breathing normally.

**Figure 11:**
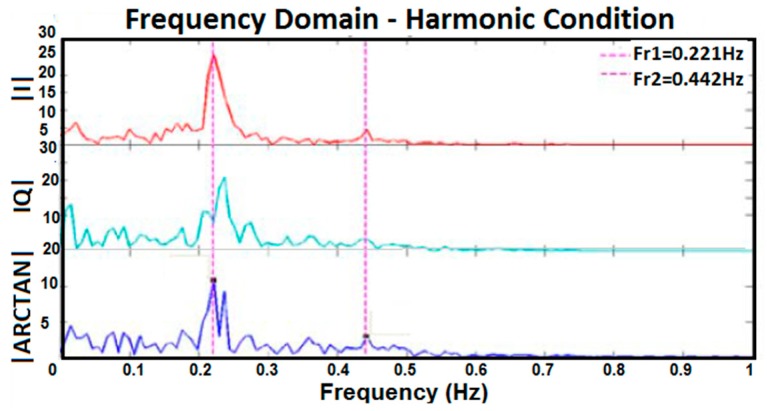
Frequency domain of I, Q, and arctangent demodulated signals for two subjects when the breathing rate of one person was harmonic to the other.

**Figure 12:**
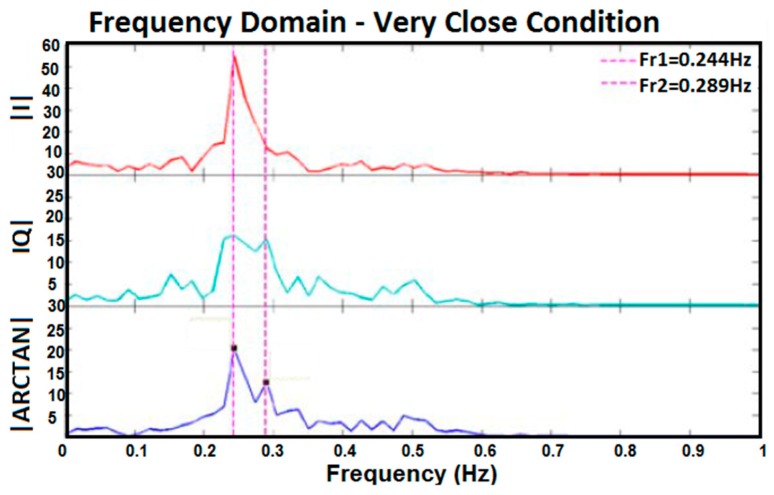
Frequency domain of I, Q, and arctangent demodulated signals for two subjects breathing too closely together.

**Figure 13:**
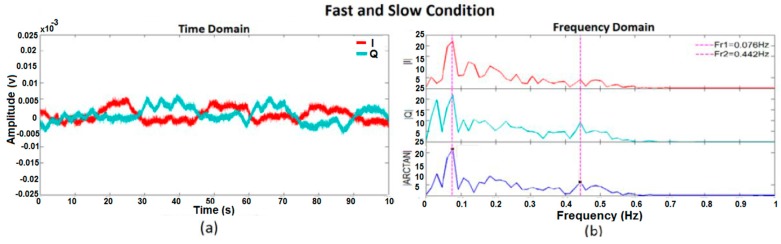
I, Q, and arctangent demodulated signals for two subjects breathing; one person too fast and the other very slow: (**a**) time domain; (**b**) frequency domain.

Figure 10, Figure 11, Figure 12 and Figure 13 show I, Q, and arctangent demodulated signals for test 3. In this case, four different situations are investigated as explained earlier. Although test 3 was performed for two subjects at the same time, similar signal-processing methods, as in Figure 5, still applied to breathing data.

The outcomes of the tests reveal if two subjects’ respiration rates are very close, or any other situation occurs which caused the amplitude of two subjects’ breathing data to be too close, the accuracy of results decreases. As shown in Figure 12, two subjects’ breathing rates are very close and, therefore, no clean frequencies can be extracted for them. Besides, if a subject’s respiration is in harmony with another subject’s, finding the accurate breathing rates of subjects will be prone to more errors, as shown in Figure 11. In Figure 11, the peak of the second person’s breathing rate is insignificant and it is difficult to differentiate it from noise. On one hand, based on time domain data and test conditions, we knew that one breathing signal was the harmonic of the other. On the other hand, the respiration rate from frequency domain is more reliable for us because it is the same in all three I, Q, and arctangent plots. As a result, a frequency was selected as breathing rate which followed both such conditions; however, the accuracy of the result was checked by the reference respiration rate of each person. The best situation in which to measure breathing rate of two subjects clearly is when the strength of breathing for both is close, as shown in Figure 10, or when they breathe at very different rates, as shown in Figure 13. In the first case, the amplitudes of both respiration rates in the frequency domain are visible and clear, and in the second case they have greater frequency differences. Again, in Figure 13, although one of the breathing rate’s amplitude is insignificant, based on time domain data where we knew one person breathed much faster than the other, and had the same frequency in all three plots, the more accurate frequency was selected as the respiration rate. When compared with the reference signal, the selected result was correct.

**Figure 14:**
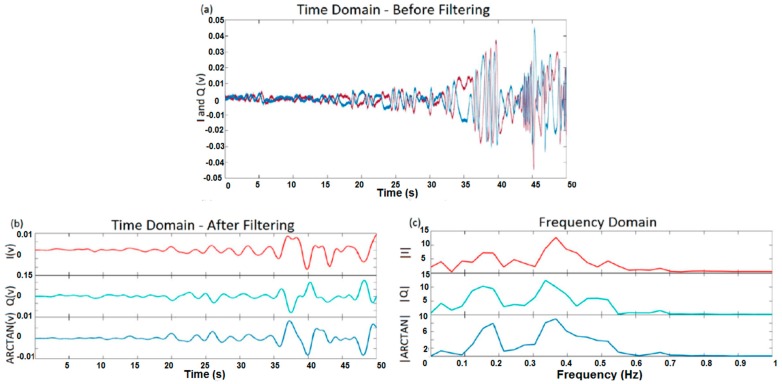
I, Q, and arctangent demodulated signals for one subject during walking: (**a**) time domain for I and Q before filtering; (**b**) time domain of I, Q, and ARCTAN after filtering; (**c**) frequency domain based on the first 25 s of signal.

For one subject, the results of test 4 are shown in Figure 14. In this figure, the I and Q outputs and the arctangent of the Q/I ratio are shown in time and frequency domains. The aim of test 4 was to determine whether or not the system had the ability to measure respiration rate during walking. In this case, because of body movement, there would be lots of irrelevant frequency components. The results demonstrate that, when a subject is very close to antennas, noisy frequency components have high amplitude. Therefore, extracting breathing frequency data is very difficult. The solution is to select a part of the signal with lower amplitude. For test 4, the first 25 s was selected. Based on the figures, the results are too noisy, yet the breathing rate of the subject is still extractable.

**Figure 15:**
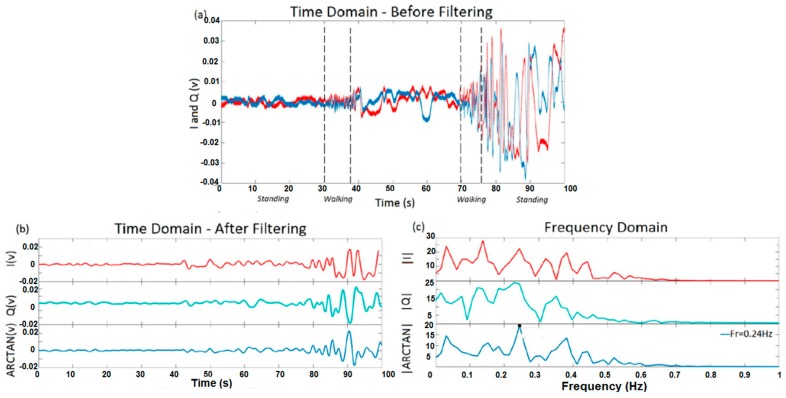
I, Q, and arctangent demodulated signals for one subject during walking and standing: (**a**) time domain for I and Q before filtering; (**b**) time domain of I, Q, and ARCTAN after filtering; (**c**) frequency domain.

Figure 15 shows I, Q, and arctangent demodulated signals for test 5. This test was designed to compare the results of respiration rate extraction from one signal in different situations as follows: walking, standing, and varying distances. The aim of test 5 was to have a different situation in each test to show how noisy the data can be before the breathing of subjects is not able to be detected. The distance of 150 cm from the antennas and an angle of 45° were the boundaries beyond which no reliable breathing data could be extracted from the signal. However, this boundary was for our setup with 15 dB transmitter power. As shown in Figure 15, the expected frequency of breathing was 0.25 Hz, but there are other strong frequency components in data. The frequency domain was extract from the first standing portion of the signal (distance of 150 cm).

## 4. Conclusions

This work discusses the design and implementation of a single Doppler radar platform with less complexity, including data-processing software to evaluate respiration rates for one or more people in varying conditions, including different distances. The experimental evaluation of the system has demonstrated that it is possible to monitor breathing of one and two subjects in varying standing and walking positions. One important aim of this study was to investigate the reliability of the system for different distances and positions (and at different angles to the source). For this purpose, five tests were done in different standing (facing and not facing the antennas) and walking situations of 30 cm, 90 cm, and 150 cm, and three angles (0°, 22.5° and 45°) in front of the antennas. The five different tests described in this paper showed that the system can measure breathing rates in standing and walking situations.

One suggestion for future work is to use information from DC components. Since DC components include information about a subject’s position and body properties, which can vary for different people, they can be used for more accurate estimation. Since such DC information includes not only a person’s position but other DC components received from hardware and other places, extracting usable DC data is a challenging future task.

As mentioned in previous sections, the maximum distance to obtain meaningful signals for the setup is 150 cm. Since one of future aims of the system is using it in normal situations of people and during ordinary activities, it will be helpful if the system can measure signals in a wider coverage range. One suggestion in this regard is using amplifiers with higher gain or using antennas with less power consumption.

One other suggestion for future work is using sensor data fusion techniques. Data fusion is the process of combining relevant information from two or more data sources into one, which provides a more accurate description than any of the individual data sources. Therefore, fusion of the data from multiple sensor sources can become a fundamental yet non-trivial task that directly impacts application performance [22]. 

## Figures and Tables

**Table 1:** Comparison of different systems.

References	P/S ^1^	NSST ^2^	NV ^3^	ST ^4^	SR ^5^ (Meter)	Technology	Frequency
[10]	Lying	1	10	HB ^6^	on Body	CW ^8^ Radar	1 GHz
[7]	Vertical: Standing/facing Antenna	1	1	RR ^7^	No Contact	CW ^8^ Doppler Radar	2.45 GHz
[4]	Standing/walking	1 and 2	1	HB ^6^	Wearable Sensor	UWB ^9^ Wireless Communication	3.5–4.5 GHz
[14]	Standing	1	1	RR ^7^	1 to 5	UWB ^9^ Radar	-
[2,15]	Sitting/Supine	1	3	HB ^6^	1	Quadrature Doppler Radar	2.4 GHz
[8]	Standing	1	1	HB ^6^/RR ^7^		Doppler Radar	2.4 GHz
[1]	Standing	1	1	HB ^6^/RR ^7^	1	CW ^8^ Radar	12 GHz/24 GHz
[9]	Standing	1	1	HB ^6^/RR ^7^	0.55	UWB ^9^ Radar/Stepped Frequency CW ^8^	3 GHz
[16]	Sitting	1	1	HB ^6^/RR ^7^	1	Doppler Radar	5.8 GHz
[11]	Lying	1	1	HB ^6^	0.5	Radar	10, 15/18 GHz
[3]	Simulation			HB ^6^		Direct Conversion Doppler Radar	2.4 GHz
[17]	Sitting	1	1	HB ^6^		Doppler Radar	5.8 GHz/20 GHz
[18]	Simulation			HB ^6^/RR ^7^	0.5, 1 & 2	Millimetre-Wave Doppler Radar	60 GHz
[19]	Walking	1	2	Target Position	5 × 5	UWB ^9^ Radar	2.45 GHz
[20,21]	Walking		Multi	HB ^6^/RR ^7^	8 × 8	Injection Locked I/Q Receivers	2.4 GHz
This Work	Standing/walking	1 and 2	8	RR ^6^	0.3–1.5	CW ^8^ Radar	4 GHz

^1^ Positioning/Situation; ^2^ Number of subjects in same test; ^3^ Number of volunteers; ^4^ Signal type; ^5^ Sensing range; ^6^ Heartbeat; ^7^ Respiration rate; ^8^ Continuous wave; ^9^ Ultra-wideband.

**Table 2:** Respiration rates (Hz) for seven subjects at the same distance from the antennas and facing in the same direction.

Colour Representing Each Subject	Respiration Rate (Hz)
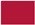	0.3052
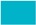	0.2441
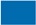	0.3586
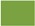	0.3357
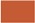	0.3662
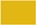	0.3967
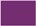	0.1984
